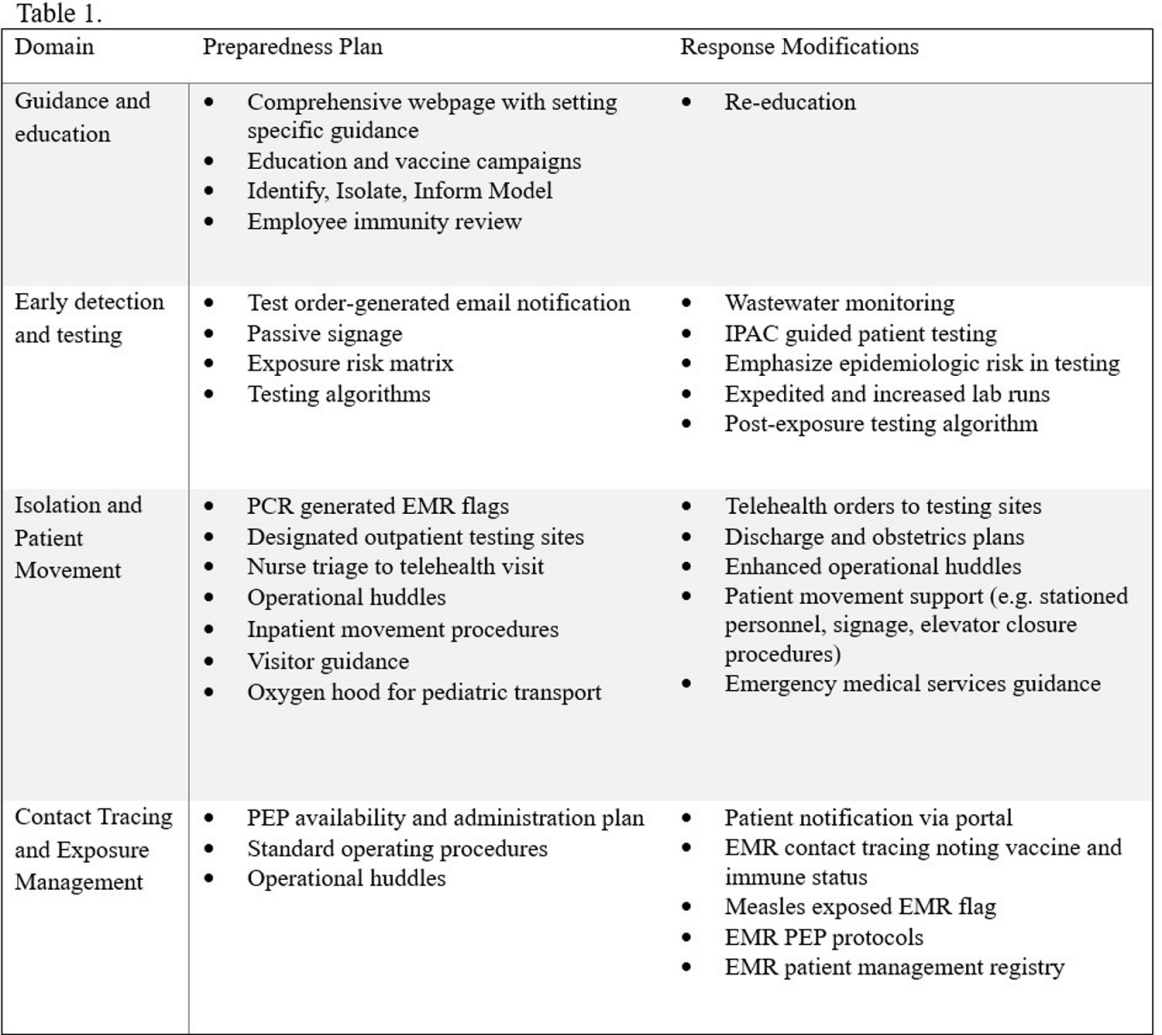# 338 Dalbavancin: National Perceptions and Experiences Among Veterans Affairs (VA) Providers

**DOI:** 10.1017/ash.2026.10679

**Published:** 2026-06-23

**Authors:** Leah Siple, Jean Barth, Rebecca Faller, Alyssa Olson, Jenna Rasmusson, Michelle Meyer, Becky Baxter, Emily Loberg, Leah Higbe, Debra Apenhorst, Sarah Bellows Mahler, Elena Beam, John OHoro, Emily Levy, W. Charles Huskins, Priya Sampathkumar

**Affiliations:** 1 Mayo Clinic; 2 Mayo; 3 Mayo Clinic Rochester; 4 Mayo Clinic College of Medicine; 5 Mayo Clinic, Rochester, MN

## Abstract

**Background:** Advanced planning and adaptable response strategies are essential for preventing measles transmission in healthcare facilities. Objective: To describe a measles management plan and subsequent modifications in response to a cluster of measles cases. **Methods:** Drawing on previously published work (doi:10.1017/ice.2025.49), the Infection Prevention and Control (IPAC) team initiated a multi-disciplinary team and developed a measles preparedness plan with the following domains: Guidance and education; early detection and testing; isolation and patient movement; contact tracing and exposure management (Table 1). In October 2025, a patient was admitted during their fifth healthcare encounter and was diagnosed with measles. Two family members were later hospitalized with measles. Delayed recognition of the first case prompted enhancements to our preparedness framework (Table 1). **Results:** The first case resulted in 539 exposed patients. The second resulted in 87 exposures, and the third resulted in no exposure. Despite the extensive exposure, zero secondary measles were reported. **Conclusion:** Proactive planning enabled rapid response to our first case and controlled secondary transmission. Despite extensive planning, delayed identification of the first case caused significant exposures. As the cluster unfolded, we refined our plan, resulting in zero exposures from the last patient and zero secondary cases, despite over 850 exposures. These efforts may guide other healthcare facilities in improving their measles preparedness and response.

**Figure:**